# Supplementary material for: NERVE 2.0: boosting the new enhanced reverse vaccinology environment via artificial intelligence and a user-friendly web interface
Source: BMC Bioinformatics. 2024 Dec 18;25:378. doi: 10.1186/s12859-024-06004-0 (PMC11654298; doi:10.1186/s12859-024-06004-0)
Supplement: Supplementary file 1 — Additional file 1. NERVE 2.0 supplementary material, with theoretical background of the adopted ML model performance measures [file 12859_2024_6004_MOESM1_ESM.pdf]

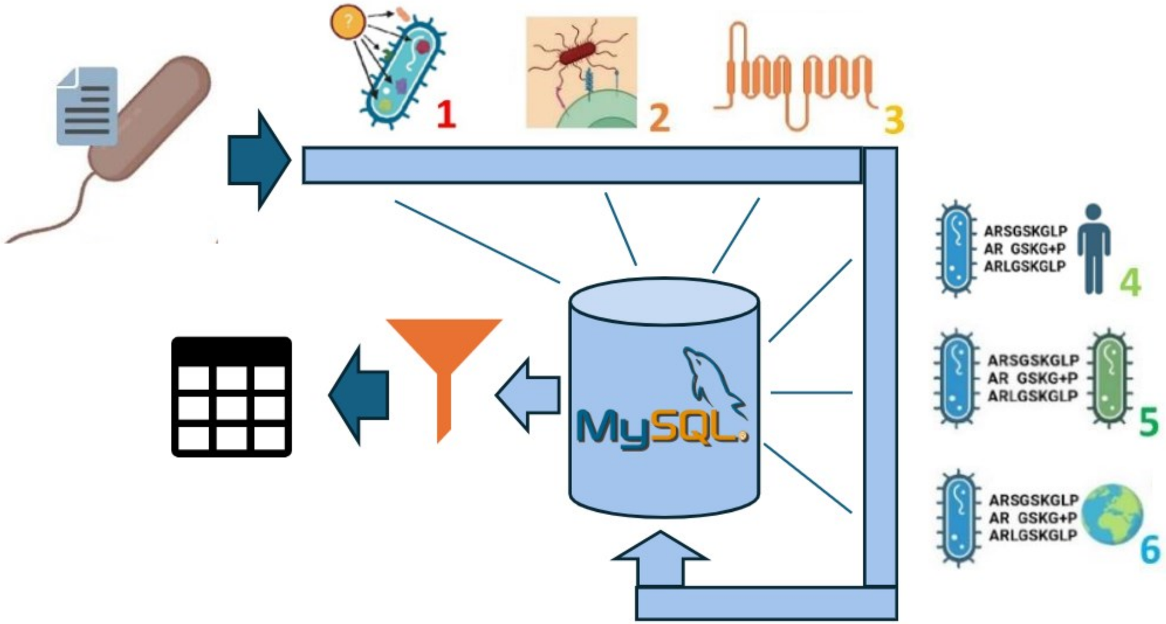

Additional figure 1. Original NERVE pipeline. Protein sequences from an input proteome are analysed through six steps, including predictions of: (1) subcellular sorting by PSORTb [5], (2) adhesin probability by SPAAN [6], and (3) membrane topology by HMMTOP [7]. Moreover, (4-5-6) sequence comparison applications based on BLASTp [8] are used to identify conserved epitopes and determine inter-strain conservation. Then, the collected information is saved in a MySQL table and finally filtered by the last module, named *select*. Extracted PVCs are listed in an HTML table. Created with BioRender.com.
