# Supplementary material for: NERVE 2.0: boosting the new enhanced reverse vaccinology environment via artificial intelligence and a user-friendly web interface
Source: BMC Bioinformatics. 2024 Dec 18;25:378. doi: 10.1186/s12859-024-06004-0 (PMC11654298; doi:10.1186/s12859-024-06004-0)
Supplement: Supplementary file 2 — Additional file 2. NERVE 1.0 pipeline, with related caption describing all tools involved and its overall working scheme [file 12859_2024_6004_MOESM2_ESM.pdf]

Additional file 2

NERVE 2.0: boosting the New Enhanced Reverse Vaccinology Environment via artificial intelligence and a user-friendly web interface

Model performance measures

To assess the performances of the two newly-developed neural networks (ESPAAN and Virulent), for each of them, a confusion matrix was created and the most common evaluation metrics were directly inferred from it. Additionally, a k-fold cross validation was used to evaluate how the models work with new unseen data. Therefore, in this section, a brief theoretical background of these mentioned model performance measures is reported.

1. Confusion Matrix

A confusion matrix can be defined as an organized way to summarize the performance of a ML (machine learning) model [1], showing which of its predictions are correct and which incorrect for all classes. In this specific case, each model classifies its input in two distinct classes.

In fact, both ESPAAN and Virulent have to solve a binary classification problem. The first model classifies input-analyzed proteins into adhesins or not-adhesins; Virulent, instead, predicts if a protein is a virulence factor or not.

Since dealing with two binary classifiers, we created two bidimensional confusion matrices. A common "2x2" confusion matrix, reported as an example, is shown in Figure 1.

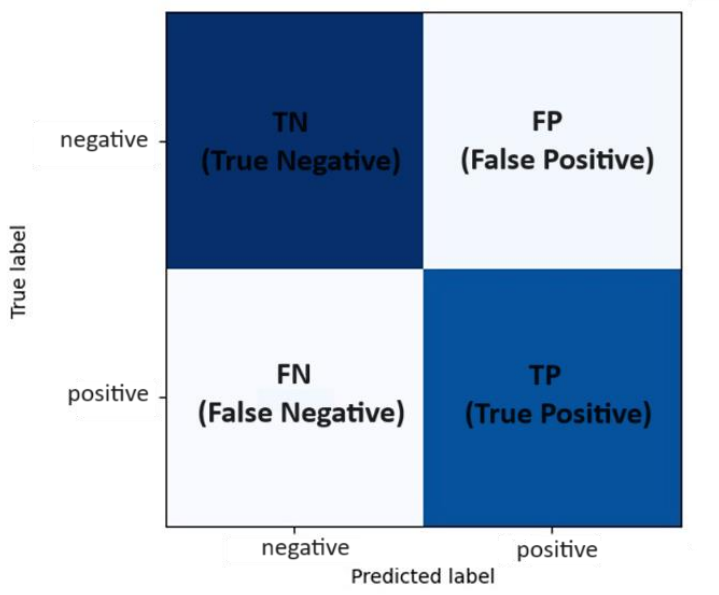

Figure 1: Example of a 2x2 confusion matrix

As evidenced, there are four different values (one for each matrix cell), which are useful to understand if the tested model is making predictions which deviate from real values, or not, and how many times this happens.

It is necessary to underline that these real values come from the known datasets we used to train our binary classifiers (supervised machine learning).

The values mentioned in Fig. 1 are:

- TN (True Negative), which is the number of real negative samples that the tested model classifies as negative.
- TP (True Positive), which represents the number of real positive samples correctly classified.
- FP (False Positive), which is the number of real negative samples, classified as positive.
- FN (False Negative), which is the number of real positive samples, classified as negative [1,2].

So, in the case of ESPAAN, TN is the number of known non-adhesins which are correctly identified by ESPAAN as non-adhesins. Instead, FP in Virulent represents the number of known non-virulent proteins which are classified as virulent.

From these values, it is possible to compute the model evaluation metrics.

2. Evaluation metrics

The evaluation metrics are crucial to assess the effectiveness of ML models, being quantitative measures of their performance [2]. All their values are expressed with a number between 0 and 1. We used nine different metrics here explained.

One of the most used metrics for classification is accuracy, which is calculated with the following formula:

$$Accuracy = (TP + TN) / (TP + TN + FP + FN) \quad (1)$$

It's a fundamental metric representing the number of all correct predictions out of all predictions. So it shows the overall correctness of the ML model [2].

Another important metric is the precision, also called Positive Predictive Value (PPV). It shows the correctness of all the positive predictions, so it is the portion of TP among all model positive

$$\boldsymbol{Precision = TP / (TP + FP)} \quad (2)$$

predictions [2] and it is defined as:

Recall instead, known also as sensitivity or True Positive Rate (TPR), is the model's ability to find all the positive samples in a dataset and it is expressed as the ratio between TP and all actual positives (TP+FN) [2].

$$Recall = TP / (TP + FN) \quad (3)$$

The harmonic mean between precision and recall values, obtained by combining them, is known as F1-score [2].

A low F1-score often indicates an imbalanced performance of the ML model. On the other hand, a F1-score closer to 1 indicates that the model, with high recall and high precision, is really good at recognizing both positive and negative samples. This score can be computed adopting this formula:

$$\boldsymbol{F1 - score \;=\; (2\, x\, precision\, x\, recall)\, /\, (precision \;+\; recall)} \qquad (4)$$

Another considered evaluation metric is Specificity or True Negative Rate (TNR). As shown by the following formula, it is the portion of TN that was correctly identified in the chosen dataset.

$$\boldsymbol{Specificity \;=\; TN\, /\, (TN \;+\; FP)} \qquad (5)$$

This measure describes how well a ML model can identify TN [2].

Instead, Negative Predictive Value (NPV) is the portion of TN among all model negative predictions.

$$\boldsymbol{NPV \;=\; TN\, /\, (TN + FN)} \qquad (6)$$

This value can also be seen as the opposite of PPV, or precision. Indeed, precision measures the TP fraction over all positive predictions, instead NPV is focused on TN detection from all negative predictions [3].

The last three reported measures can be derived from some of the just analyzed metrics. Different from the latters, when evaluating a good-performing model, low values of these rates are expected.

The False Positive Rate (FPR) is calculated as the ratio between FP and all actual negatives.

$$\boldsymbol{FPR \;=\; FP\, /\, (FP + TN)} \qquad (7)$$

It can be also calculated with this formula: $FPR \;=\; 1 \;-\; TNR$ (or specificity) (8) [3].

The False Negative Rate (FNR), also called miss-rate, is expressed as the ratio between FN and all actual positives.

$$FNR \;=\; FN\, /\, (FN + TP) \qquad (9)$$

Another usable formula for FNR is : $FNR \;=\; 1 \;-\; TPR$ (or recall) (10) [3].

Lastly, False Discovery Rate (FDR), is the fraction of FP over all positive predictions and it can be obtained as follows:

$$\boldsymbol{FDR \;=\; FP\, /\, (FP + TP)} \qquad (11)$$

FDR is also complementary to PPV, or precision. Indeed, it can be expressed as: $FDR \;=\; 1 - PPV$ (12) [3].

Evaluation metrics values for ESPAAN and Virulent are listed and commented respectively on 2.2 and 2.8 paragraphs in the article.

3. k-fold cross validation

K-fold cross validation is a specific type of cross validation (CV) technique, which is widely used to test ML models performance. In particular, it can be really helpful in avoiding overfitting, an undesired situation which occurs when a ML model has great accuracy with training data but not with test data. Then, k-fold CV is useful to estimate the model generalization, and so its ability to do appropriate predictions adapting to new input data yet to be tested [4].
As shown in Figure 2, the dataset is divided into *k* approximately equal parts or “folds”.

The tested model is trained *k* times using a different fold as the validation set and the remaining *k-1* folds as the training set. This way, every data point in the dataset is used once for validation and *k-1* times for training. The value of *k* can vary, with common values being 2, 3, 5, 10, or even 20. The value is empirically chosen, and ideally, the best value is the one that minimizes both the bias and the variance of the model.

In our approach, we aimed to keep a reduced model complexity by diminishing the number of parameters. Consequently, we expected a high bias and low variance in our results. Achieving strong performance in both training and validation phases—using our established Keras validation framework for training the current models—we opted for k = 3 to ensure our models ability to generalize. This choice implies that each iteration's training set closely reflects the original, in cardinality. Increasing *k* would result in smaller training sets per iteration, potentially compromising the models learning process [6][7].

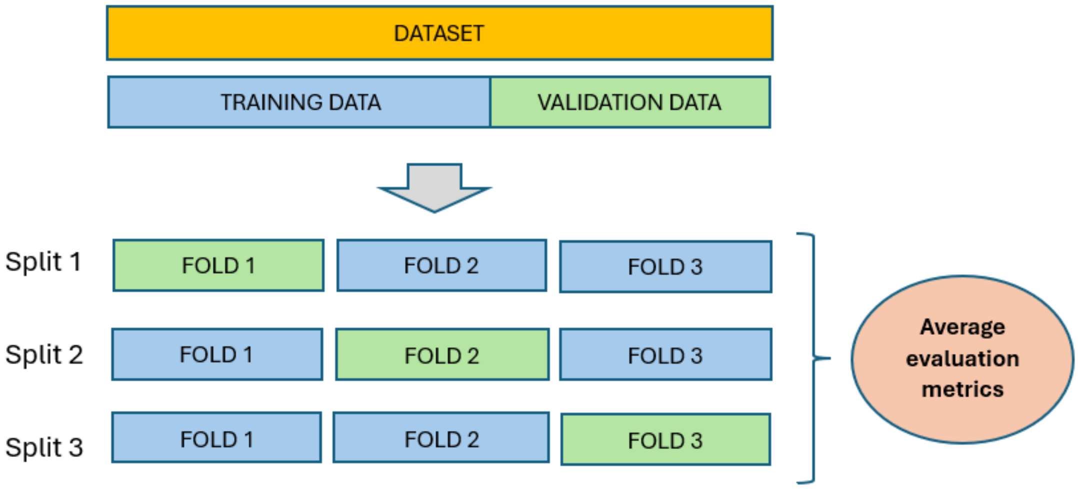

Figure 2: Working scheme of k fold cross validation with k=3

For each split, the principal model performance measures are computed and the score of the entire procedure is determined by averaging the *k* resulting values [4].
The relative standard deviations have to be computed, as well as the mean values.

Standard deviation (σ) is a fundamental statistical tool that measures the dispersion or the variability of a dataset, intended here as a set of measures, around the mean value. It is also useful to compare the variability of datasets having different units of measurement [5]. A smaller value indicates less

variability, while a larger one indicates greater variability. In our case, we used the standard deviation to quantify the error/uncertainty connected to model measures, specifically the CV mean evaluation metrics.
It is defined as the square root of the variance (σ^2), which is calculated using the following formula:

$$(\boldsymbol{\sigma^2}) = \boldsymbol{\Sigma(xi - \mu)^2}/n \quad (13)$$

Where *Σ* represents summation, *xi* is each data point, *μ* is the mean of the dataset and *n* is the dataset size (number of data points) [5].
So, the standard deviation is expressed as:

$$\boldsymbol{\sigma} = \sqrt{\boldsymbol{\Sigma(xi - \mu)^2}/n} \quad (14)$$

Even if the variance is a dispersion data measure as the standard deviation, it is not generally expressed in the same units as the dataset and doesn't have smaller values than the second one. All this makes the standard deviation an easier interpretable measure of data dispersion [5].

All k-fold cross-validation mean values and the related standard deviations are reported and discussed in the 2.2 and 2.8 paragraphs of the article.

References

1. Ajay Kulkarni, Deri Chong and Feras A. Batarseh. 5 - Foundations of data imbalance and solutions for a data democracy. Data Democracy. Academic Press. 2020; Pages 83-106
2. Hossin, M. and Sulaiman, M.N. A Review on Evaluation Metrics for Data Classification Evaluations. International Journal of Data Mining & Knowledge Management Process, 2015 5, 11 p.
3. Richard G. Brereton. False discovery rates, power and related concepts. Journal of Chemometrics. 2020 May; Volume 35, Issue 6.
4. Farhad Maleki, Nikesh Muthukrishnan, Katie Ovens, Caroline Reinhold and Reza Forghani. Machine Learning Algorithm Validation: From Essentials to Advanced Applications and Implications for Regulatory Certification and Deployment. Neuroimaging Clinics of North America. 2020; 30, 4, 433-445
5. El Omda S, Sergent SR. Standard Deviation. StatPearls. Treasure Island (FL). 2024 Jan
6. Max Kuhn, Kjell Johnson. Applied Predictive Modeling. Springer New York. 2013 May
7. Gareth James, Daniela Witten, Trevor Hastie and Robert Tibshirani. An introduction to Statistical Learning.Springer New York. 2021 July
